# Using clinical decision support to improve urine testing and antibiotic utilization

**DOI:** 10.1017/ice.2023.30

**Published:** 2023-10

**Authors:** Michael E. Yarrington, Staci S. Reynolds, Tray Dunkerson, Fabienne McClellan, Christopher R. Polage, Rebekah W. Moehring, Becky A. Smith, Jessica L. Seidelman, Sarah S. Lewis, Sonali D. Advani

**Affiliations:** 1 Division of Infectious Diseases, Department of Medicine, Duke University School of Medicine, Durham, North Carolina; 2 Duke Center for Antimicrobial Stewardship and Infection Prevention, Durham, North Carolina; 3 Duke University School of Nursing, Durham, North Carolina; 4 Continuous Improvement Department, Duke University Health System, Durham, North Carolina; 5 Clinical Microbiology Laboratory, Duke University Health System, Durham, North Carolina; 6 Department of Pathology, Duke University of Medicine, Durham, North Carolina

## Abstract

**Objective:**

Urine cultures collected from catheterized patients have a high likelihood of false-positive results due to colonization. We examined the impact of a clinical decision support (CDS) tool that includes catheter information on test utilization and patient-level outcomes.

**Methods:**

This before-and-after intervention study was conducted at 3 hospitals in North Carolina. In March 2021, a CDS tool was incorporated into urine-culture order entry in the electronic health record, providing education about indications for culture and suggesting catheter removal or exchange prior to specimen collection for catheters present >7 days. We used an interrupted time-series analysis with Poisson regression to evaluate the impact of CDS implementation on utilization of urinalyses and urine cultures, antibiotic use, and other outcomes during the pre- and postintervention periods.

**Results:**

The CDS tool was prompted in 38,361 instances of urine cultures ordered in all patients, including 2,133 catheterized patients during the postintervention study period. There was significant decrease in urine culture orders (1.4% decrease per month; *P* < .001) and antibiotic use for UTI indications (2.3% decrease per month; *P* = .006), but there was no significant decline in CAUTI rates in the postintervention period. Clinicians opted for urinary catheter removal in 183 (8.5%) instances. Evaluation of the safety reporting system revealed no apparent increase in safety events related to catheter removal or reinsertion.

**Conclusion:**

CDS tools can aid in optimizing urine culture collection practices and can serve as a reminder for removal or exchange of long-term indwelling urinary catheters at the time of urine-culture collection.

Urine cultures obtained in the absence of clinical signs or symptoms do not provide actionable information in most patient populations because they identify a high prevalence of asymptomatic bacteriuria.^1^ Positive urine cultures in catheterized patients are difficult to interpret due to high rates of colonization and contamination during specimen collection, and they have a low predictive value for true infection.^2^ Indwelling urinary catheters are colonized at a rate of 3%–7% per day.^3,4^ Nonspecific clinical signs and symptoms, such as fever, often trigger urine-culture orders in hospitalized patients with indwelling urinary catheters. In addition, subjective findings, such as color or odor of urine or sediment in tube, influence nurses and providers to overorder urine cultures in catheterized patients.^5^ In many instances, clinicians may not even be aware that the patient has a urinary catheter in place when ordering a urine culture.^6^

Overuse and misuse of urine cultures in catheterized patients leads to inappropriate antibiotic use and artificially inflates the diagnosis of catheter-associated urinary tract infections (CAUTIs).^1,5,7^ Hence, Infectious Diseases Society of America (IDSA) and Society for Healthcare Epidemiology of America (SHEA) guidelines recommend replacing long-term urinary catheters before urine specimen collection.^8,9^ However, guidance on optimal timing of catheter exchange at the time of urine collection is unclear. Prior studies have examined periods ranging from 24 hours to 14 days for catheter replacement at the time of urine collection.^3,10,11^ These interventions, however, have primarily focused on the outcomes of surveillance CAUTI, without measuring the impact on antibiotic use or unintended consequences like catheter trauma.^3,10–13^

Clinical decision support (CDS) tools can assist with appropriate urine testing and collection techniques, can reduce diagnostic error, and can improve antibiotic use. We evaluated the effect of a CDS tool on health system–level urine-culture volume, antibiotic utilization for urinary tract infection (UTIs), and catheter use, and we examined safety signals related to catheter trauma among patients who required a catheter exchange.

## Methods

### Study design

In this before-and-after intervention study, we examined the impact of CDS tool launched in March 2021 as part of a quality improvement initiative. The study was divided into 2 periods: a 12-month preintervention period (March 2020–February 2021) and a 12-month postintervention period (April 2021–March 2022). The study was considered exempt by Duke University Institutional Review Board (no. 00108749).

### Setting and population

This study was conducted in 3 hospitals in North Carolina: 1 academic medical center, Duke University Hospital (1,048 inpatient beds), and 2 community hospitals, Duke Raleigh Hospital (175 beds) and Duke Regional hospital (388 beds). All emergency room visits and inpatient admissions of any age were eligible to prompt the CDS tool. Different base populations were utilized within this cohort to analyze the outcomes outlined below.

### Intervention

**Fig. 1.**
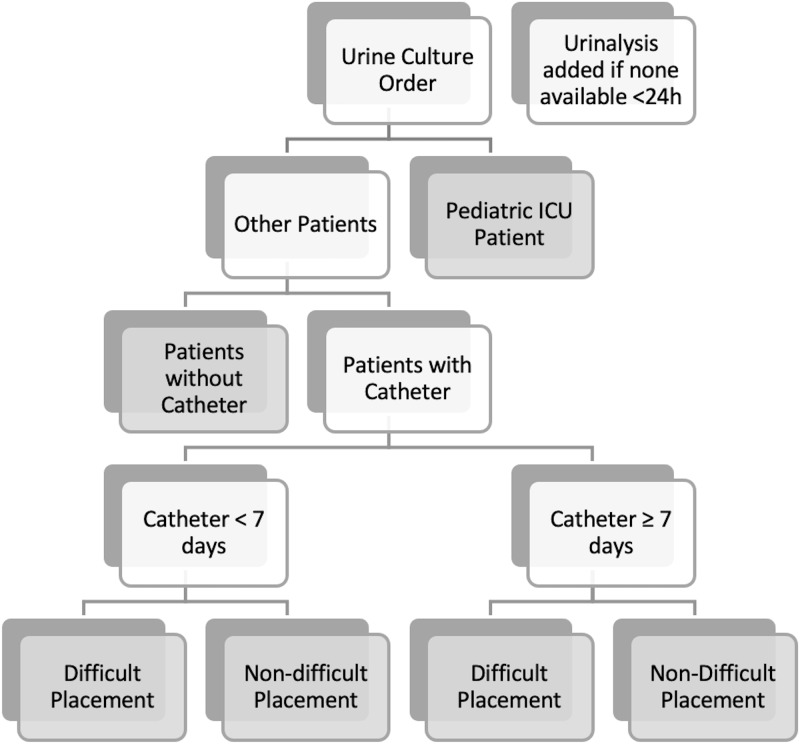
Branching logic to identify appropriate patient populations for panel display. Gray boxes represent a branch-point ‘terminus’ that has unique decision support dependent on the population identified.

The CDS tool was designed to provide education on appropriate indications for urine culture in all patients regardless of catheter presence. It uses patient-specific data to prompt catheter exchange when needed, incorporates information from prior urine tests, and ultimately reduces the volume of inappropriate urine cultures. The panel was developed with input from the institutional infection prevention and antimicrobial stewardship teams, pediatrics, urology, and infectious disease clinicians. Four primary features were implemented within the clinical decision support panel: (1) passive educational information; (2) branching-logic identification of specific patient populations based on coded criteria (ie, patients with indwelling urinary catheters stratified by duration of existing catheter placement, pediatric intensive care patients (PICU)); (3) an adjustable list of urine-culture or nursing orders with the ‘recommended’ action based on the identified patient population listed first; and (4) identification of an existing or pending urinalysis order or result within 24 hours, with a prompt that defaults adding a urinalysis order if none is found (Fig. 1). When a clinician ordered a urine culture in a patient with an indwelling urinary catheter, the order panel provided education regarding appropriate clinical indications for a urine culture and recommended catheter removal prior to urine culture for indwelling urinary catheters in place for >7 days, after excluding PICU patients and catheters with difficult placement (Supplementary Fig. 1 online). We chose 7 days as a time frame that would limit harm from rapid removal or replacement of urinary catheters but would not allow for significant colonization of long-term indwelling catheters prior to urine culture compared to alternative time frames (ie, <24–48 hours after placement or up 14 days after placement). Finally, the intervention was also designed to limit additional ‘clicks’ in the electronic health record (EHR). In fact, for the most commonly encountered clinical scenarios, the intervention actually reduced the number of clicks required to order a urine culture.

### Outcomes

The primary outcome was urine culture utilization, measured as urine cultures per 1,000 patient days among all hospitalized patients. Secondary outcomes included urinalyses per 1,000 patient days, days of antibiotic therapy with urinary tract infection (UTI) indication per 1,000 patient days, and urinary catheter standardized utilization ratio (SUR) among all hospitalized patients. We also measured safety events related to catheter insertion as catalogued by event reports in the Duke Safety Reporting System (SRS). Outcomes measured among catheterized patients included number of catheter removal orders and catheter-associated urinary tract infections (CAUTIs) per 1,000 catheter days.

### Definitions

A CAUTI was defined according to the Centers for Disease Control and Prevention (CDC) National Healthcare Safety Network (NHSN) surveillance criteria.^14^ The SUR was defined as the number of observed device days reported compared to predicted based on the NHSN model.^15^ Antibiotic therapy for urinary tract infection (UTI) indication was defined as any hospital-administered antibiotic that had an electronically entered indication category of “genitourinary” or subcategories of “uncomplicated urinary tract infection,” or “complicated urinary tract infection” at the time of the order.

### Data collection

Electronic order-entry data and laboratory result data were extracted from the Duke Epic Clarity data warehouse. Antibiotic use data were extracted, processed, and cleaned via the Duke Antimicrobial Stewardship Outreach Network central database.^16^ Antibiotic use was measured in days of therapy (DOT) per 1,000 patient days for inpatient units reported to the National Healthcare Safety Network (NHSN). Urinary catheter SUR was calculated monthly, as reported to the NSHN. Potential adverse events, including catheter trauma, were extracted via the Duke Safety Reporting System (SRS) database.

### Analysis

Outcomes were analyzed using an interrupted time-series analysis. The preintervention rate trend (March 1, 2020, through February 28, 2021) was compared to the postintervention rate trend (April 1, 2021, through March 31, 2022) using Poisson logistic regression. A difference in rate change in March 2021 was also measured, though the data points for March 2021 were excluded from regression analysis because the CDS tool was implemented partway through this month. All analyses were performed using Python version 3.7 software.

## Results

**Fig. 2.**
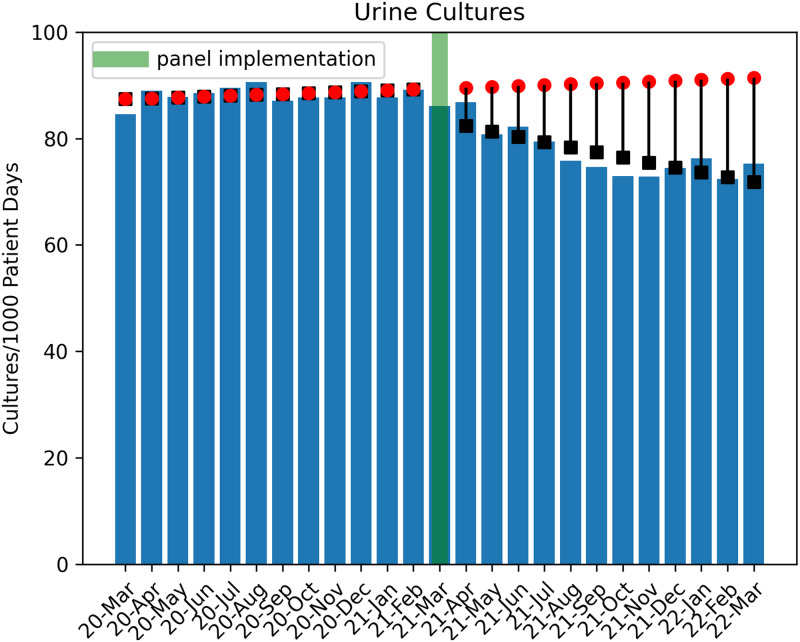
Trend of urine-culture orders in the pre- and postintervention periods. Circles indicate predicted outcome ‘without intervention.’ Boxes indicate Poisson regression model estimates.

**Fig. 3.**
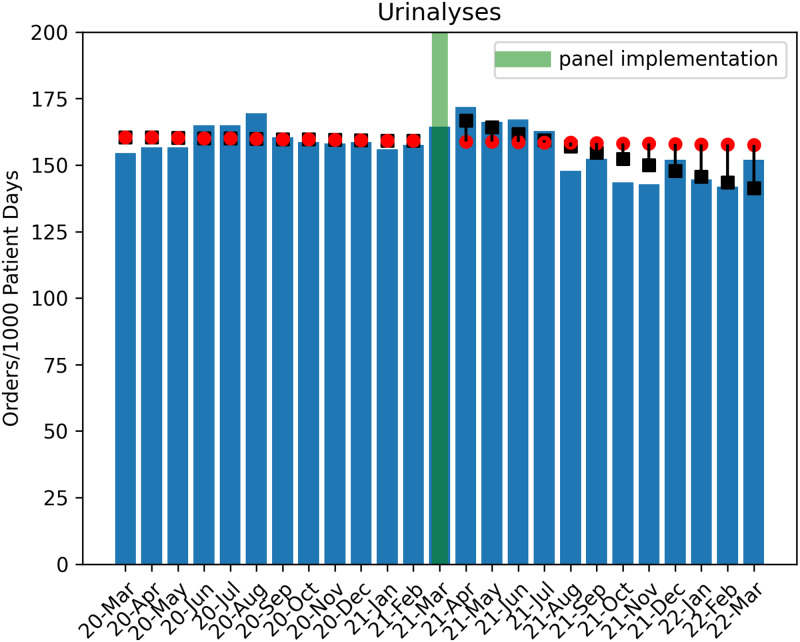
Trend of urinalysis orders in the pre- and postintervention periods. Circles indicate predicted outcome ‘without intervention.’ Boxes indicate Poisson regression model estimates.

In total, 77,608 urine culture orders and 148,694 urinalysis orders were included in the study analysis (excluding 3,511 culture orders and 5,754 urinalysis orders for March 2021). Poisson regression analysis revealed a significant decrease in urine culture orders per 1,000 patient days in the postintervention period (1.4% decrease per month; *P* < .001) (Fig. 2). During the postintervention period, there was a total estimated reduction of 6,743 urine culture orders when compared to the estimated ‘without intervention’ model. An immediate increase of urinalyses orders by 6.2% (*P* < .001) occurred, with a subsequent monthly decline of 1.4% per month (*P* < .001), which tracked with the decrease in urine cultures (Fig. 3). By the end of the postintervention period, a comparison of the ‘with’ and ‘without intervention’ models indicated a reduction in total urinalyses of 2,300 orders.

**Fig. 4.**
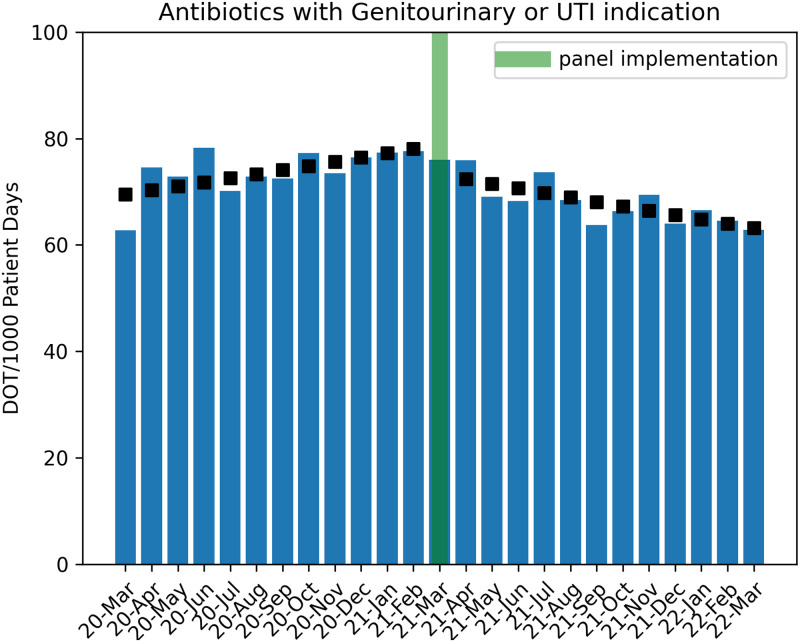
Trend of antibiotic utilization in the pre- and postintervention periods. Boxes indicate Poisson regression model estimates. Note. UTI, urinary tract infection indication; DOT, days of therapy.

Antibiotic use for UTIs was increasing by 1.1% per month in the preintervention period. At the time of panel intervention in March 2021, there was an immediate decrease of 7.1% in antibiotic use for UTIs, followed by a decreasing monthly trend of ∼2.3% DOT per 1,000 patient days in the postintervention period (*P* = .006) (Fig. 4). During the urine-culture order-entry process, when a catheter was detected (of any duration) by the panel logic, clinicians selected catheter removal in a total of 183 (8.5%) of 2,133 instances. The SUR for urinary catheters and number of CAUTIs did not change significantly during the study period (Table 1 and Supplementary Figs. 1 and 2 online).

**Table 1.** Impact of Clinical Decision Support on Urine Test Utilization and Other Outcomes in the Pre- and Postintervention Periods

Interrupted Time-Series Models with Poisson Regression				
Variable	Intercept	Baseline Trend RR (95% CI)	Trend Change RR (95% CI)	Level Change RR (95% CI)
Urine Cultures (Urine cultures/1,000 patient days per month)	86.287 (83.044–89.657)	1.002 (0.9999–1.005)	**0.986 (0.982–0.990)**	**0.933 (0.906–0.962)**
Urinalyses (Urinalyses/1,000 patient days per month)	161.397 (156.876–166.048)	0.999 (0.997–1.001)	**0.986 (0.983–0.989)**	**1.064 (1.042–1.087)**
Antibiotic use with GU or UTI Indication (DOT/1,000 PD per month)	64.592 (61.684– 67.636)	**1.011 (1.007–1.014)**	**0.977 (0.973–0.982)**	**0.929 (0.897–0.962)**
CAUTIs (CAUTIs/1,000 Foley catheter days per month)	0.057 (0.210–1.555)	1.049 (0.976–1.128)	0.956 (0.869–1.051)	0.900 (0.468–1.734)
SUR (SUR rate per month)	0.706 (0.224–2.221)	1.000 (0.900–1.111)	0.995 (0.798–1.241)	1.038 (0.168–6.414)

Note. RR, rate ratio; CI, confidence interval; DOT, days of therapy; PD, patient days; CAUTI, catheter-associated urinary tract infection, GU, Geintoruinary; UTI, urinary tract infection; SUR, standardized utilization ratio. Bold indicates *P* value < .05.

An evaluation of Duke Safety Reporting System (SRS) identified 3 events during the study period related to orders for removal or replacement or a urinary catheter. Of these 3 events, 2 occurred in the preintervention period and 1 event occurred in the postintervention period. The event in the postintervention period was related to catheter insertion and was not associated with an order for catheter exchange from the urine-culture order panel.

## Discussion

Clinical decision support for ordering urine cultures was associated with a decrease in overall utilization of urine cultures across our health system. The logic-based decision support also provided a simple, direct method for clinicians to identify and remove or exchange long-term indwelling catheters prior to obtaining a urine culture. Following the implementation of this CDS, we also observed a decrease in antibiotic use with UTI indications. Additionally, no instance of urethral trauma was reported from replacement of longstanding catheters at or after day 7 using our logic.

Prior literature has shown that a computerized CDS tool can improve inpatient antimicrobial utilization and contribute to diagnostic stewardship.^17^ Although some prior research highlighting CDS relied on urine culture indications as entered by clinicians, a process which increases the steps (or ‘clicks’) an end-user must take to order a test, we implemented a panel that added no additional workload to the ordering clinician.^18^ Our findings of decreased diagnostic test utilization (ie, urine culture and urinalysis) and UTI-specific antibiotic use are consistent with other studies using CDS at time of order entry.^18–20^ Because the majority of tests ordered during the study period were for patients without a urinary catheter in place, our significant outcomes of decreased test utilization and antimicrobial stewardship were likely a result of the passive, educational, decision-support component of the implemented order panel. Literature describing CDS for catheter removal in the context of urine culture ordering is limited. Frontera et al^3^ reported that a protocol requiring catheter removal at the time of urine sampling led to a significant reduction in CAUTI rates. However, this protocol involved replacing catheters in place for 24 hours at the time of urine sampling, without measuring unintended consequences like catheter trauma.^3^ In our initiative, we opted to allow for a longer duration of indwelling catheters before prompting removal to limit clinical scenarios requiring catheter replacement and potential trauma. This longer duration likely decreased a potential impact on CAUTI rates because catheter removal was not as strongly prompted within the 7-day period.

To our knowledge, this study is the first description of implementation of a branching-logic CDS panel for the urine-culture order process. A primary goal of this quality improvement initiative was to provide real-time education and relevant patient-centered clinical information while also streamlining the order process and reducing overall clicks required from clinicians to achieve the original desired outcome (urine culture order). Streamlining workflow in the EHR is often difficult to achieve in stewardship efforts that frequently require hard stops or additional clicks such as indication fields on antimicrobials or diagnostic tests. The results of this study may indicate the beginning of a sustained culture shift of ordering fewer urine cultures for patients, which may result in lower antimicrobial use with UTI indications while avoiding the frustration inherent with the addition of new steps in EHR workflows. The branching-logic features also allowed clinicians to identify long-term indwelling catheters and to prompt removal in safe and appropriate populations as identified by pediatric and adult infectious diseases and urology specialists.

Although catheter removal was only selected in a fraction of patients with indwelling catheters (8.5%), catheter removal was not the original ‘intent’ of the clinician when entering the urine culture order as prior to the logic-based pane implementation, and catheter removal would not have been part of the urine-culture order workflow. Prior data have shown that >20% of clinicians may not be aware that their patient has an indwelling urinary catheter.^6^ Hence, our CDS intervention also serves as a subtle reminder to remove the catheter in these instances when a urine culture is being ordered but the clinician is either unaware of the presence of the catheter or has not assessed the ongoing need.

Our study had several limitations. It was implemented in a single health system. The before-and-after intervention analysis was subject to time-associated confounding, including other stewardship efforts to reduce inappropriate urine-culture ordering and antibiotic use for suspected UTIs. In addition, we were able to evaluate the total number of instances that a clinician ordered catheter replacement when the panel detected one in place, but due to limitations in data extraction, we were not able to distinguish whether these 183 orders occurred when the catheter was in place fewer than or more than 7 days. Finally, adverse events related to catheter trauma are difficult to capture and may be underrepresented by the SRS system (both before and after the intervention).

Our study is unique in that our primary outcomes include volume of urine culture orders and antibiotic use. Additionally, our data captured unintended consequences like catheter trauma. Our interval of 7 days for prompting catheter removal or replacement may provide a more feasible time frame to balance early inappropriate removal versus unnecessary longstanding catheters compared to prior studies that have recommended replacement early (24–48 hours) or late (14 days). However, the optimal duration until prompting clinicians to remove catheters with decision support is unknown, and future studies are needed to address this knowledge gap.

Many EHR-based interventions are implemented as quality improvement initiatives; thus, it is difficult to disentangle the effect of multiple features of a decision support process that are simultaneous. Future studies that evaluate decision support for urine cultures in a randomized, controlled manner could identify the potential efficacy and effect size of the various decision support features. However, based on the results of our before-and-after analysis of the quality improvement initiative, our CDS tool was associated with reduced urine-culture utilization and antibiotic use.

## Supplementary material

For supplementary material accompanying this paper visit https://doi.org/10.1017/ice.2023.30. click here to view supplementary material
