# Supplementary figures and images for: Using clinical decision support to improve urine testing and antibiotic utilization
Source: Infect Control Hosp Epidemiol. 2023 Mar 29;44(10):1582–6. doi: 10.1017/ice.2023.30 (PMC10539479; doi:10.1017/ice.2023.30)

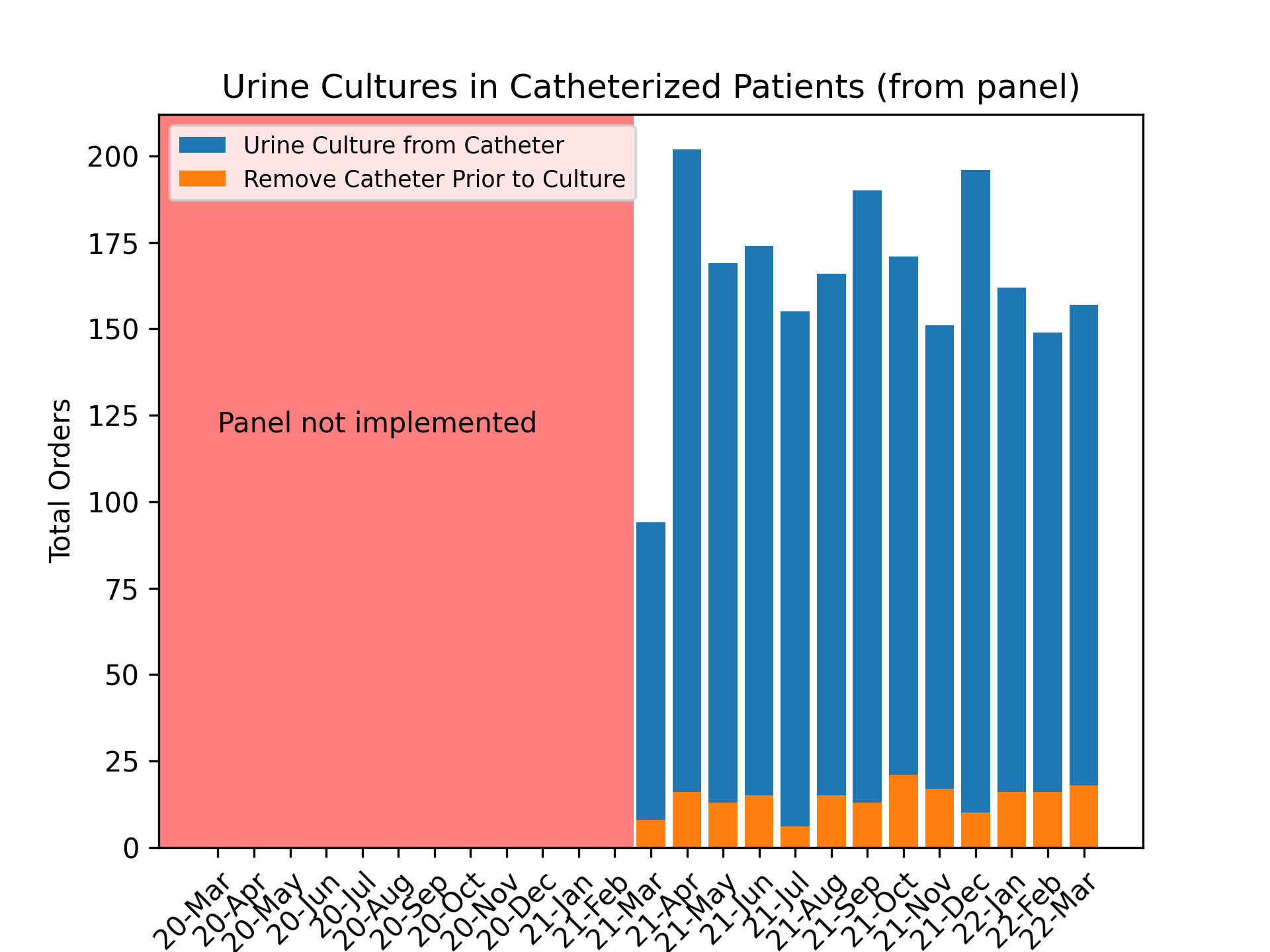

Supplement: Supplementary file 1 [file S0899823X23000302sup.zip › S0899823X23000302sup002.tiff]

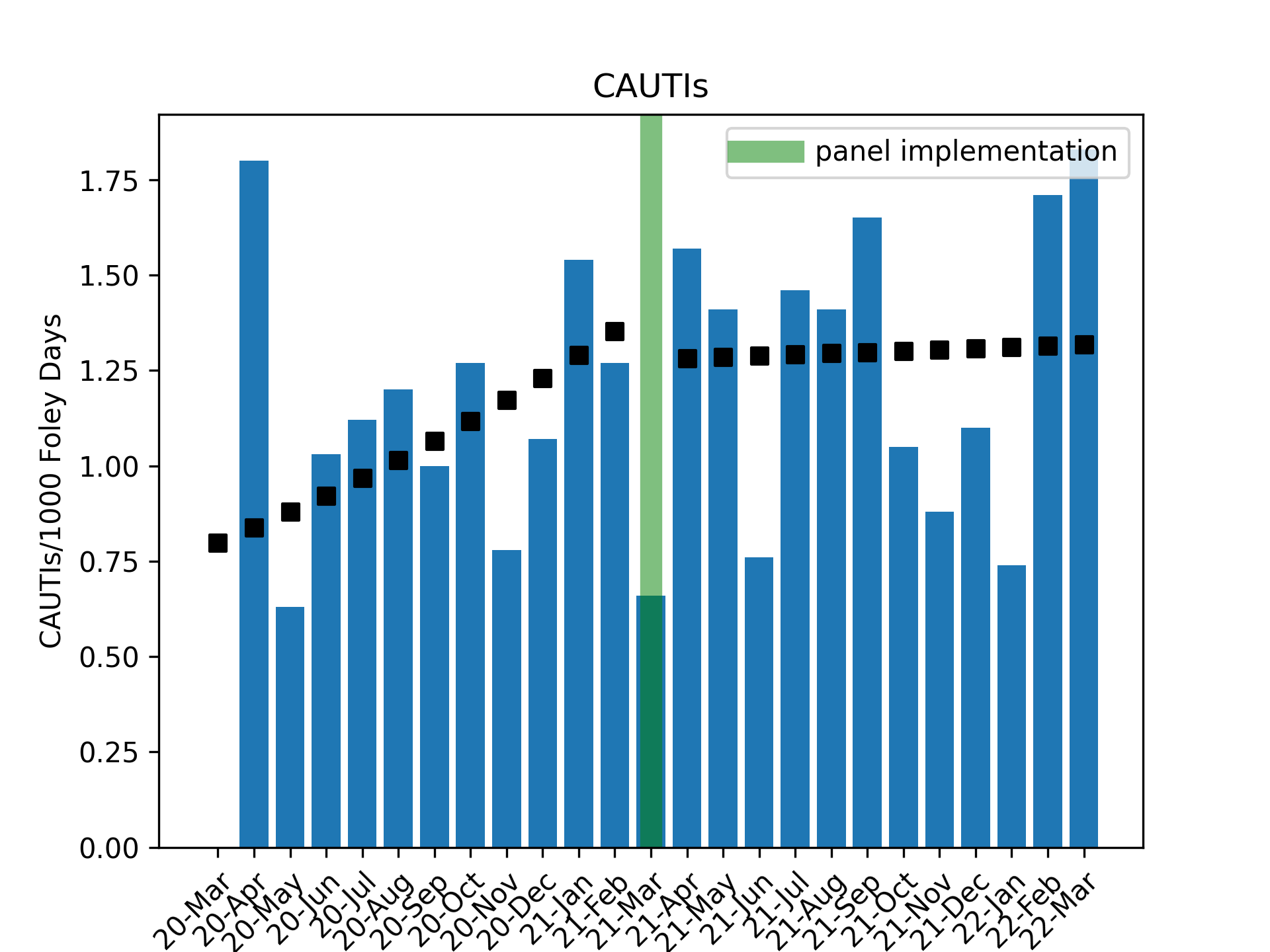

Supplement: Supplementary file 1 [file S0899823X23000302sup.zip › S0899823X23000302sup003.tiff]
